# Plant traits database for climate change adaptation and mitigation in Northwest Portugal

**DOI:** 10.1016/j.dib.2022.108193

**Published:** 2022-04-20

**Authors:** Catarina Patoilo Teixeira, Cláudia Oliveira Fernandes, Jack Ahern, Paulo Farinha-Marques

**Affiliations:** a InBIO - Rede de Investigação em Biodiversidade e Biologia Evolutiva, CIBIO, Universidade do Porto, Campus Agrário de Vairão, Vairão 4485-661, Portugal; b Departamento de Geociências, Ambiente e Ordenamento do Território, Faculdade de Ciências, Universidade do Porto, rua do Campo Alegre 687, Porto 4169-007, Portugal; c BIOPOLIS Program in Genomics, Biodiversity and Land Planning, CIBIO, Campus de Vairão, Vairão 4485-661, Portugal; d Department of Landscape Architecture and Regional Planning, University of Massachusetts, Amherst, MA 01003-2901, USA

**Keywords:** Climate change adaptation, Climate change mitigation, Green infrastructure, Plant traits, Planting design, Urban ecological novelty, Urban green spaces, Porto

## Abstract

The database presented in this data article is related to the article “*Adaptive planting design and management framework for urban climate change adaptation and mitigation*” [1]. It includes a list of 287 plant species presently occurring in Porto, Portugal, more precisely in urban green spaces with high urban ecological novelty levels. The plant species in this list were classified and organized according to several traits with a particular focus on plant species’ adaptation, mitigation, and ornamental characteristics. Data collection resorted to articles, books, and various open access and online datasets. Data were organized in an Excel file that organizes information on more than 50 plant species traits/variables.

## Specifications Table

**Table:** 

Subject	Biodiversity
Specific subject area	Plant traits for climate change adaptation and mitigation in Northwest Portugal
Type of data	Table Figure Excel database
How data were acquired	Literature search of published data
Description of data collection	Data was collected from published literature and also through open access and online plant traits datasets. Plant species were searched by scientific name. Specific traits considered climate change adaptation and mitigation, and ornamental value.
Data source location	See “Trait source data” in https://data.mendeley.com/datasets/p7y8yv7psn/1
Data accessibility	Repository name: Mendeley Data Data identification number (permanent identifier, i.e. DOI number): https://doi.org/10.17632/p7y8yv7psn.1 Direct link to the dataset: https://data.mendeley.com/datasets/p7y8yv7psn/1
Related research article	C.P. Teixeira, C.O. Fernandes, J. Ahern, Adaptive planting design and management framework for urban climate change adaptation and mitigation, Urban For. Urban Green. (2022). 10.1016/j.ufug.2022.127548.

## Value of the Data

• Compilation of data from various publications and databases about plant traits that have an active role in climate change adaptation and mitigation and ornamental value.
• The data is useful to researchers interested in studying plant traits and landscape design and management practitioners interested in applying the compiled knowledge.
• The data can assist the design and/or management of plant communities in cities for climate change adaptation and mitigation goals, and also considers plants’ ornamental value.
• The data facilitates the selection of plant species for all types of urban green spaces.
• The data represent a starting point and the database can continue to be developed, so other relevant species and traits can be included over time as knowledge about climate change adaptation and mitigation increases.

## 1 Data Description

**Fig. 1:**
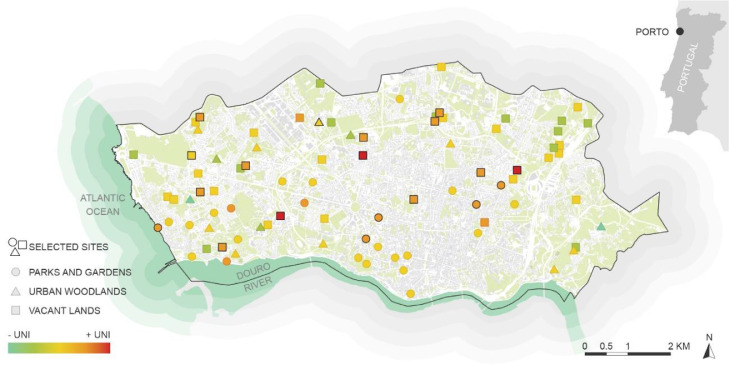
Level of urban ecological novelty throughout 85 urban green spaces in Porto, Portugal. 19 sites highlighted in the figure selected based on a higher Urban Ecological Novelty Index (UNI).

This work presents a plant traits database that will assist the design or management of plant communities in cities for climate change adaptation and mitigation, considering as well the ornamental value of plants. The database is available online in Mendeley Data (https://data.mendeley.com/datasets/p7y8yv7psn/1). In total, the dataset includes available trait information for 287 plant species from 75 botanical families and 206 genera. Fig. 1 shows the locations where the plant list was retrieved. Table 1 lists the traits included in the database and respective data sources.

**Table 1:** List of traits selected to organize the database. Main focus: A – Adaptation, M – Mitigation, and O – Ornamental.

Trait	Classes	Main focus
PLANT ID & DISTRIBUTION		
Botanical name & authorship		
Common name (EN)		
Common name (PT)		
Family		
Genera		
Status	Native, non-native, non-native with ecological risk (casual, naturalized, or invasive)	A
Risk and gravity analysis	Low risk, medium risk, high risk (for casual and naturalized) Low gravity, medium gravity, high gravity (for invasive)	A
Geographical distribution	Cosmopolitan, Eurasia and North Africa, Europe, outside Europe, Mediterranean and Macaronesia, endemic, uncertain origin	A
PLANT FITNESS & TOLERANCE		
Light	Full sun, partial, full shade	A
Light plasticity	Number of light types covered: from 1 to 3	A
Soil substrate	Sandy, loamy, clayey	A
Soil substrate plasticity	Number of soil substrate types covered: from 1 to 3	A
Soil pH	Acid, neutral, alkaline	A
Soil pH plasticity	Number of soil pH types covered: from 1 to 3	A
Soil moisture	Dry, fresh, moist	A
Soil moisture plasticity	Number of soil moisture types covered: from 1 to 3	A
Temperature hardiness zone	Hardiness zones range	A
Temperature hardiness plasticity	Number of zones covered: from 2 to 10	A
Known tolerances and sensitivities	Drought, heat, maritime exposure, pollution, flooding, wind	A
PLANT STRUCTURE		
Habit	Tree, shrub, subshrub, herb/forb, grass/sedge, fern, climber, bamboo, palm	M, O
Life form	Hydrophyte/helophyte, geophyte, therophyte, hemicryptophyte, chamaephyte, nanophanerophyte, microphanerophyte, mesophanerophyte, megaphanerophyte	A, M, O
Growth rate	Slow, moderate, fast	M
Height and Width	Expected height and width (categories in meters)	M, O
Shape	Clumped/tufted, columnar, oval, pyramidal, round, spreading, umbrella, vase/weeping	M, O
Crown density*	High, medium, low	M, O
Multi-stem development	Yes, no	M, O
Foliage color	Green, Green-yellowish, Green-reddish, Green-purplish, Green-bluish, Green-greyish	O
Foliage fall color*	No fall color, Yellow, orange, red, purple, brown	O
Foliage persistence*	Deciduous, evergreen, semi-evergreen	M, O
Flower color	White, cream, yellow, orange, red, pink, purple, blue, green, brown, inconspicuous	O
Flower bloom time	Months range (Jan-Dec)	A, O
Flower bloom time plasticity	Number of bloom months covered: from 1 to 12	A, O
Fruit/seed color	White, cream, yellow, orange, red, pink, purple, blue, green, brown, inconspicuous	O
OTHER FEATURES		
Known functions	Erosion control, fragrant parts, phytoremediation, shading, windbreak, nitrogen fixer, wildlife resources (birds and insects)	M, O
Known hazards	Allergy or toxicity, invasive risk, odor nuisance, thorns or spikes	M, O

⁎ Information only for trees and shrubs.

## 2 Experimental Design, Materials and Methods

### 2.1 Plant species selection

The first step in the elaboration of this database comprised the selection of a list of plant species. For that, we resorted to a previous work that assessed level of urban ecological novelty throughout 85 urban green spaces in Porto (Fig. 1), belonging to three different urban green spaces categories: Parks and Gardens, Urban Woodlands, and Vacant Lands [2], [3], [4]. We selected 19 sites (out of a total of 85 urban green spaces) where the level of urban ecological novelty was higher, based on the Urban Ecological Novelty Index (UNI). The 19 sites are highlighted in Fig. 1 and established a list of 287 plant species.

### 2.2 Traits’ selection and data collection

The list of 287 plant species was the starting point for the development of this database. Following that step, we selected a list of traits based in core landscape planting publications and with a particular focus on adaptation, mitigation, and ornamental characteristics [5], [6], [7], [8], [9], [10], [11], [12], [13], [14]. Data was collected in several publications (articles and books) and also in open access and online databases.

Traits were organized in four categories:

• Plant ID & distribution – refers to information that supports the identification of the plant species. This group also has information about the plant origin (nativeness) and distribution range, which is very relevant information under climate change.
• Plant fitness & tolerance – refers to information about the species fitness, tolerance, and plasticity (ability to perform across a range of environmental conditions) to different environmental conditions (light, soil, temperature, or water).
• Plant structure – refers to information about the whole plant structure (life form, shape, height and width, etc.) and also about the characteristics of particular plant parts (foliage, flower, and fruit).
• Other features – refers to other important features of plants for climate change adaptation and mitigation, but also to improve ornamental value and human well-being and safety.

To each trait we defined a comprehensive list of classes, allowing a straightforward classification and organization of all plant species in the database. We included all the available information we were able to find, still a small portion of the plant species in the database lack information regarding some traits.

Information about the selected traits and respective classes are detailed in Table 1. Information about the traits source references is detailed and available online in Mendeley Data (https://data.mendeley.com/datasets/p7y8yv7psn/1).

## CRediT authorship contribution statement

**Catarina Patoilo Teixeira:** Conceptualization, Methodology, Investigation, Visualization, Writing – original draft. **Cláudia Oliveira Fernandes:** Conceptualization, Methodology, Writing – review & editing, Supervision. **Jack Ahern:** Conceptualization, Writing – review & editing, Supervision. **Paulo Farinha-Marques:** Resources.

## Declaration of Competing Interest

The authors declare that they have no known competing financial interests or personal relationships which have or could be perceived to have influenced the work reported in this article.

## Data Availability

Supplementary Material: Plant traits database for climate change adaptation and mitigation in Northwest Portugal (Reference data) (Mendeley Data).
